# Prevalence and hepatic histopathological findings of fascioliasis in sheep slaughtered in Jeddah, Saudi Arabia

**DOI:** 10.1038/s41598-023-33927-0

**Published:** 2023-04-24

**Authors:** Safinaz J. Ashoor, Majed H. Wakid

**Affiliations:** 1 Department of Diagnostic Laboratory, Fakieh Poultry Farm, Jeddah, Saudi Arabia; 2 Department of Medical Laboratory Sciences, Faculty of Applied Medical Sciences, King Abdulaziz University, P. O. Box 80324, Jeddah, 21589 Saudi Arabia; 3 Special Infectious Agents Unit, King Fahd Medical Research Center, Jeddah, Saudi Arabia

**Keywords:** Parasite host response, Medical research, Signs and symptoms, Diseases, Infectious diseases, Microbiology, Infectious-disease diagnostics, Parasitology, Pathogens

## Abstract

Hepatic fascioliasis is an important parasitic disease responsible for morbidity and mortality in many domestic ruminants, especially sheep, goats, and cattle, due to *Fasciola* (*F.*) *hepatica* and *F. gigantica*. This study aimed to determine the prevalence of fascioliasis in sheep slaughtered in Jeddah, Saudi Arabia, and to describe the morphological and histopathological changes in the liver. A total of 109,253 sheep slaughtered between July 2017 and July 2018 were screened to assess the prevalence of fascioliasis. The livers were grossly investigated for *Fasciola* infection and morphological changes. Tissue samples were collected for proper histopathological examinations. Livers of local and imported sheep represented infection rates of 0.67% and 2.12%, respectively, and the highest infection rate was in the spring season. Macroscopically, the affected liver showed hepatomegaly, thickened capsule and discoloration with necrosis, fibrosis, dilation of the bile duct, engorgement of the gallbladder and enlargement of the portal lymph nodes. Microscopic examination showed fibrotic thickening, calcification and hyperplasia of the bile ducts filled with debris, as well as massive hemorrhagic foci. Histopathological examinations of the infected liver showed a central vein region with disturbed parenchyma cells, focal lymphocytic infiltration, elongated endothelial cells, blood sinusoids that showed enlarged Kupffer cells, patches of lysed or necrotic hepatocytes, eosinophil infiltration, lymphocytes and proliferating fibroblast, thickening of hepatic artery and arteriolar walls. We concluded that fascioliasis among sheep slaughtered in Jeddah is not uncommon. The identified histopathological changes in the liver of infected sheep reflect tissue damage, which can lead to significant economic losses for the animals.

## Introduction

Parasitic infections are considered a serious health problem with high prevalence spread throughout most developing countries^1^. Worldwide, the liver flukes (*Fasciola hepatica* and *F. gigantic*a) are the most important ruminant parasites^2^, leading to zoonotic fascioliasis, which affects about 700 million herbivorous domestic animals^3^.

Three billion US$ or more is the annual loss due to fascioliasis (or fasciolosis) in farm animals as a sequence of productivity losses^4^. Economically, more than one contributing factor can lead to losses including medication costs, meat condemnation, lower wool quality, lower milk production, lower calf birth weight, lower overall growth rate, and increased susceptibility to secondary infections^5,6^.

For several decades, fascioliasis was considered a secondary disease in humans until 1990, when many cases were reported, and it was considered a regional disease in many countries^7^. Currently, about 17 million human cases have been reported as positive fascioliasis while about 91 million cases are suspected to be at risk of infection mainly in the Middle East, Asia, many parts of the USA, South Africa, and parts of Europe^8–13^.

*Fasciola hepatica* is widespread but is abundant in temperate and cold regions with higher altitudes in the tropics and subtropics while *F. gigantica* is found mostly in the tropics. The geographical distribution of *F. gigantica* and *F. hepatica* depends largely on the presence of suitable intermediate hosts (snails) in the environment^11^.

The oral route of infection gives clear possibilities of how the infection can be transmitted from animals to humans. Cattle, sheep, and goats can harbor infection after ingestion of encysted metacercariae in aquatic plants or they may be suspended in the soil or even in drinking water^14^. Next, the cysts travel through the intestinal wall and liver tissue until they reach the preferred habitat, the bile ducts.

The body wall of flukes plays an important role in protection, gaseous exchange, and circulating waste, and aids in the uptake of amino acids. It consists of a thick layer of cuticle, made of a homogeneous layer of scleroprotein (called the syncytial tegument) that covers the fluke and protects it from the juices of the host. It bears small spines, spinules or scales, which anchor the fluke to the bile duct, providing protection and facilitating locomotion. The innermost layer is a thin delicate basement membrane followed by a sub-cuticular musculature of three smooth muscle layers (circular, longitudinal and diagonal muscle fibers). Below the musculature is the parenchyma, which contains many loosely arranged uninucleate and bi-nucleate cells with syncytial network of fibers with fluid-filled spaces that aid in the transport of nutrients and waste products^15–18^.

Fascioliasis is characterized by chronic, sub-acute or acute inflammation of the infected liver and bile ducts. As a sequel of improper liver functions and cirrhosis, submandibular edema appears followed by anemia and anorexia ending in general intoxication and death in severe cases for prolonged periods^19^. Considering the larger flukes of *F. gigantica*, it can detrimentally suppress the host's immune system and malfunction many body functions making it more pathogenic and lethal^20^.

Accurate early diagnosis is imperative for effective control, particularly to prevent drug resistance. Traditionally, fecal examination techniques for detections of parasites eggs have been commonly used. Recently, immunologic techniques have been employed for the detection of the parasite coproantigens and serological ELISA for the detection of antibodies in blood samples. Moreover, advanced molecular identification appears to offer the most promise for the diagnosis of the infection^21–24^.

Despite the accumulated knowledge about fascioliasis in recent years and the available technological advances, we still face critical limitations. Whether it is the histological changes of the disease, the constraint treatment options or the increased resistance of the worm in both animals and humans alike, further research is required.

Therefore, this work aims to determine the prevalence of fascioliasis in Jeddah, Saudi Arabia and to investigate the gross and microscopic histological changes of infected liver in slaughtered sheep.

## Materials and methods

### Animals and study area

A total of 109,253 sheep were slaughtered in the main slaughterhouse in Jeddah (western region of Saudi Arabia). The prevalence of fascioliasis of imported and local slaughtered sheep was determined through visits every three weeks to the main slaughterhouse in Jeddah for a full year (July 2017–July 2018).

### Morphological identification and morphometric analysis

Adult flukes of *Fasciola* species are flattened, leaf-like in shape with narrow anterior and posterior ends, bilaterally symmetrical, and have no body cavity. Eight morphological parameters were analyzed to identify the adult worms. The body length and width, cone length and width, oral sucker maximum and minimum diameter, and ventral sucker maximum and minimum diameter^25–27^.

### Examination of slaughtered sheep

After skinning the slaughtered sheep, the outer surface of the carcass was examined by visual observation for any abnormal lesions and then the abdominal cavity was opened to check for the internal organs. The liver was examined from each sheep by making multiple deep incisions in the lobes and the gallbladder was opened using a knife. Observations were confirmed by a licensed and qualified veterinarian throughout inspection visits to the slaughterhouse.

### Histopathological preparation of liver tissues

Slaughtered sheep livers (normal and infected) were cut into small cubes of about 1 cm^3^ and fixed in 10% (v/v) neutral buffered formalin for 24 h. Fixed samples were washed with tap water for 12 h and then dehydrated in ascending grades of alcohol (70–100%), respectively. Tissue specimens were cleared by xylene, infiltrated in liquid paraffin wax then embedded in clean wax to be blocked. The blocked tissues were incised into 4–5 µm thick sections using a rotary microtome. The cut sections were flattened in a water bath, picked up with glass slides, and dewaxed in descending grades of alcohol (100%, 95% and 70%), respectively. Stained with hematoxylin and eosin (H&E) using standard histological protocols, or Masson Trichrome stain for fibrosis, then dehydrated in alcohol, cleared in xylene, and mounted with DPX for microscopic examination^28^.

### Statistical analysis

All collected data were reviewed, coded, tabulated, and statistically analyzed using a software package (IBM® SPSS® Statistics, SA, version 25). Data were submitted and appropriate tests were performed for each parameter, and *p*-value of < 0.05 is considered significant.

## Results

### Infection rate

**Figure 1:**
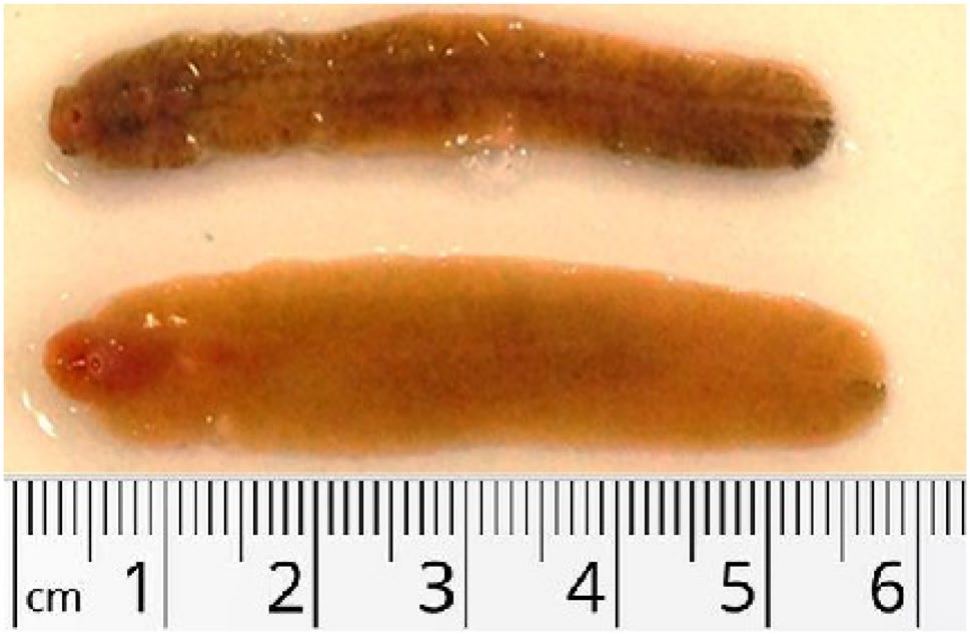
Dimension of isolated liver flukes.

After studying 109,253 sheep (36,440 local and 72,813 imported) slaughtered in Jeddah over a full year, the infection rate for local sheep was 0.67% compared to 2.12% for imported sheep (Table 1). Based on the sizes shown in Fig. 1 and Table 2, the isolated flukes are assumed to be *F. gigantica*.

**Table 1:** The number and rate of infection of local and imported sheep.

Season	Month	Number of slaughtered sheep		Number of infected sheep		Rate of infection (%)		*p1*
		Local	Imported	Local	Imported	Local	Imported	
Spring	March	2770	5960	27	161	0.97	2.70	0.098
	April	2700	4649	41	198	1.52	4.26	
	May	2521	5062	32	104	1.27	2.05	
Total		7991	15,671	100	463	1.25	2.95	
Summer	June	1870	4100	24	93	1.28	2.27	0.002
	July	1450	5000	11	132	0.76	2.64	
	August	2500	5240	16	158	0.64	3.02	
Total		5820	14,340	51	383	0.88	2.67	
Fall	September	3830	5680	19	140	0.50	2.46	0.461
	October	1971	4038	20	104	1.01	2.58	
	November	4031	6020	14	110	0.35	1.83	
Total		9832	15,738	53	354	0.54	2.25	
Winter	December	4718	8402	8	92	0.17	1.09	0.581
	January	5008	12,042	13	94	0.26	0.78	
	February	3071	6620	16	156	0.52	2.36	
Total		12,797	27,064	37	342	0.29	1.26	
All seasons	Total	36,440	72,813	241	1542	0.67	2.12	
	Mean	3036.67	6067.75	20.08	128.50	0.77	2.33	
	(SD)	1131.57	2224.88	9.50	34.36	0.44	0.89	
	*p2*					< 0.001		
	*p3*						< 0.001	
	*p4*					< 0.001		

p1, comparison between local and imported sheep during each month; p2, comparison between local infected and non-infected sheep; p3, comparison between imported infected and non-infected sheep; p4, comparison between imported and local infected sheep among all seasons.

**Table 2:** Morphometric data of the detected adult liver flukes.

Parameter	Mean ± SD (mm)
BL (Body length)	45.67 ± 5.05
BW (Body width)	9.98 ± 1.23
CL (Cone length)	3.33 ± 0.5
CW (Cone width)	3.86 ± 0.62
OS mx (Oral sucker maximum diameter)	0.90 ± 0.23
OS mn (Oral sucker minimum diameter)	0.66 ± 0.072
VS mx (Ventral sucker maximum diameter)	1.66 ± 0.15
VS mn (Ventral sucker minimum diameter)	1.54 ± 0.15

### The relationship between infection rate and season of the year

**Figure 2:**
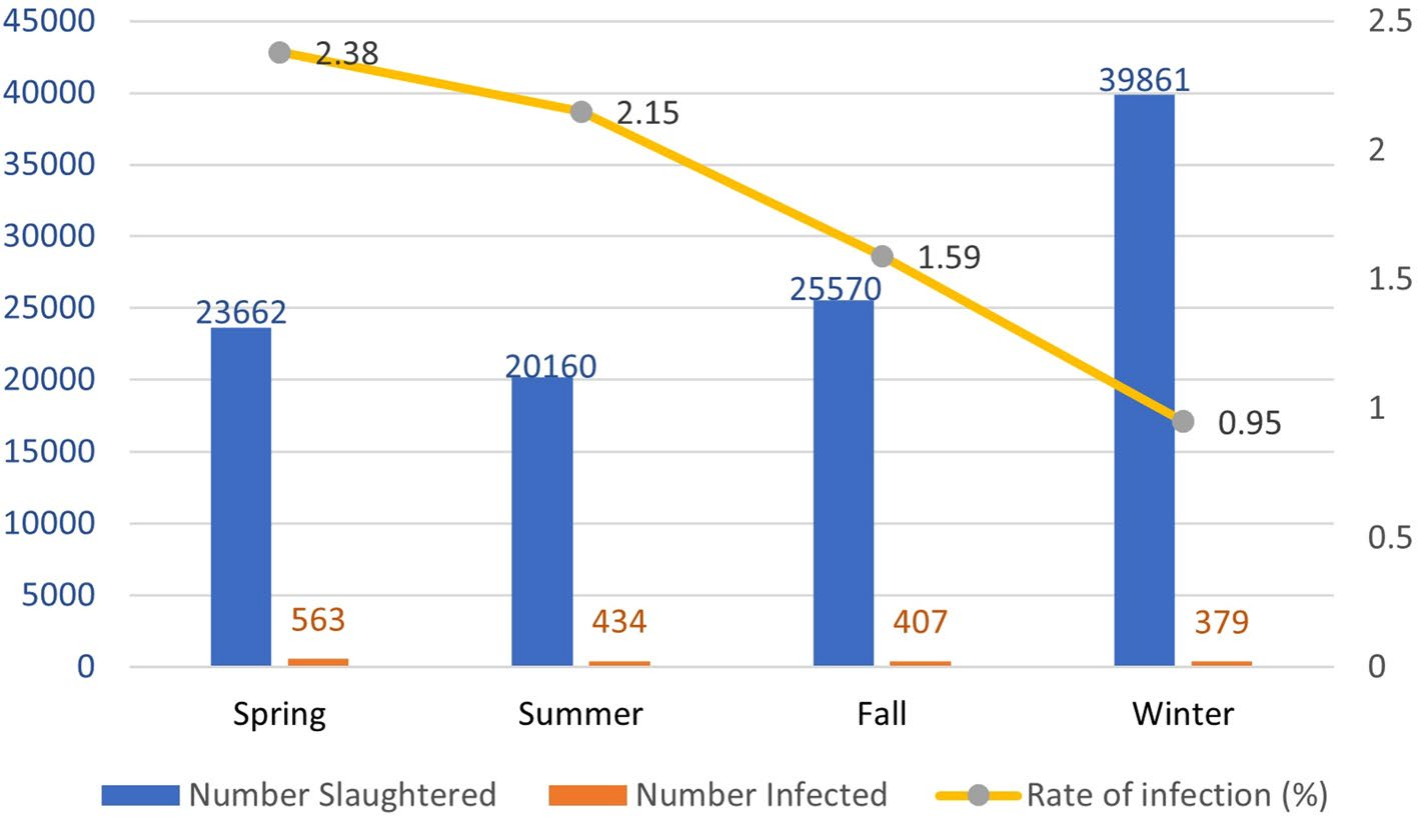
Correlation between seasons and rate of infection.

Given the span of the study through a year in the slaughterhouse, a correlation between the season when the sheep were slaughtered, and the prevalence of fascioliasis was observed. The highest infection rate was observed in spring (2.43%) followed by summer (2.15%), fall (1.59%) and then winter (0.96%), (Fig. 2).

Infection rates did not differ significantly between local and imported sheep between the months of spring (*p* = 0.098), fall (*p* = 0.461), and winter (*p* = 0.581). Among the summer months, August showed the highest rates of infection among imported sheep, while September showed the highest rates of infection among local sheep, with significant differences (*p* = 0.002).

There was descending of infection during spring, summer, fall and winter among local (*p2* < 0.001) and imported (*p3* < 0.001) infected sheep. Across all seasons, the imported infected sheep showed a higher incidence of infection than the local infected sheep, with a decreasing incidence of infection through time (*p4* < 0.001).

### Gross liver changes

In mammals, the liver is in the anterior part of the visceral cavity, behind the diaphragm. The liver was removed from sheep and morphological changes due to infection were examined and compared to normal, non-infected liver.

**Figure 3:**
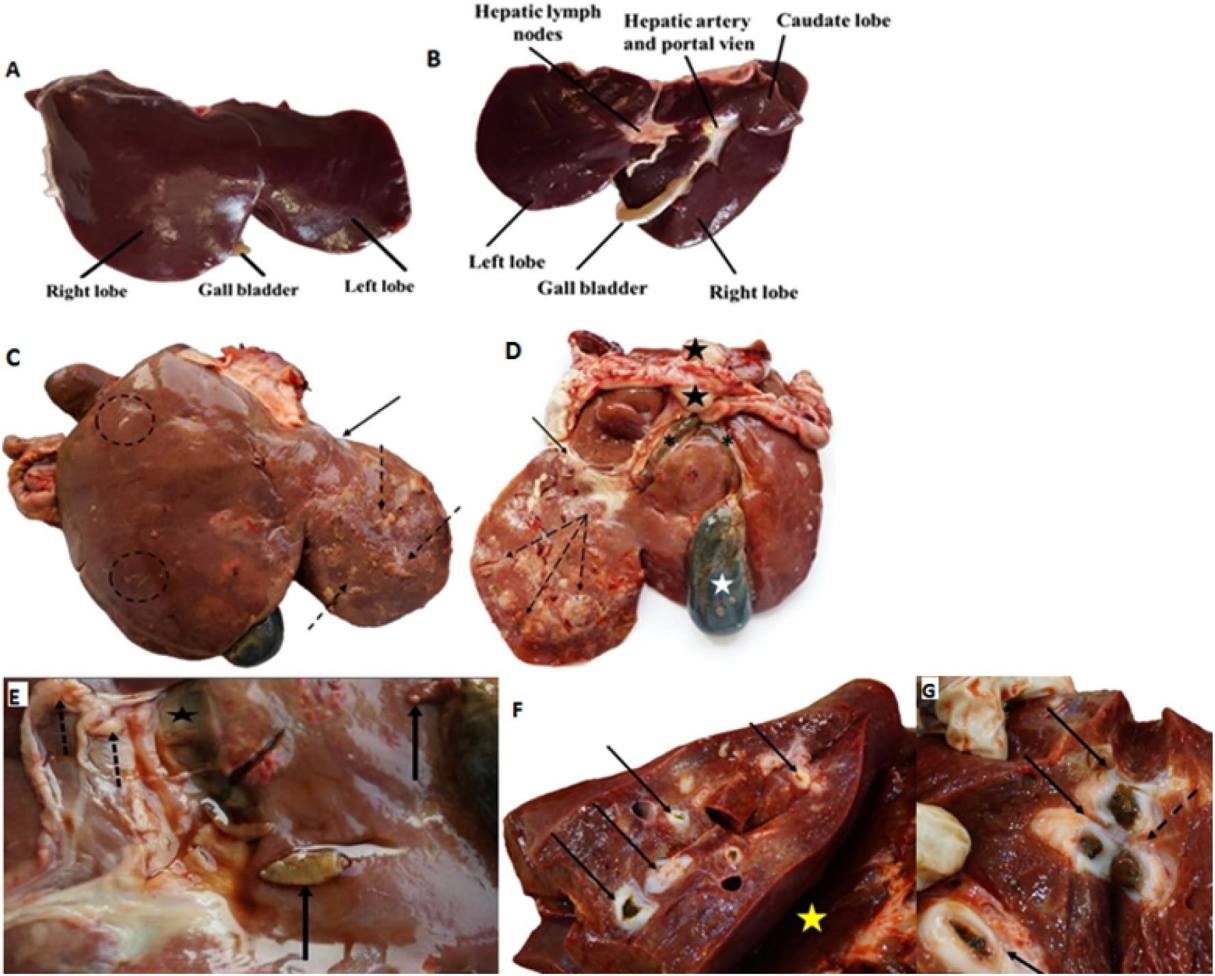
Normal liver morphology, (**A**: diaphragmatic surface view; **B**: visceral surface view). (**C**) The diaphragmatic surface of the infected liver showing lobular enlargements, thickened capsule (black arrows) and patches of fibrosis (dotted arrows). The migratory paths of worms can also be seen (dotted circles); (**D)** the visceral surface of the infected liver showing marked necrotic regions or abscess formation (dotted arrows). Note the engorgement of the gallbladder (white star), dilated bile ducts (asterisk) and enlarged portal lymph nodes (black stars); (**E**) mature and immature *Fasciola* flukes on the surface (black arrows), dilated bile ducts (black star) and enlarged portal lymph nodes (dotted arrows); (**F**) cut a section from infected liver showing fibrotic thickening and hyperplasia of the portal bile ducts (black arrows). Hemorrhagic foci (yellow star) were also presented; (**G**) cut a section from infected liver showing calcification and thickening of the bile ducts filled with debris (black arrows) and *Fasciola* fluke (dotted arrow) protruding from another bile duct can be seen.

The non-infected liver was of normal color and size with normal external surface features. The liver is divided into three lobes covered with a thin capsule of connective tissue. The gallbladder can be recognized between the two lobes (Fig. 3A and B). On the other hand, the infected liver showed external signs of liver damage confined mostly to the left lobe, the liver lobules appeared enlarged (hepatomegaly) with rounded edges and a thickened capsule. A discoloration of the liver could be seen. The surface was irregular showing patches of liver necrosis and fibrosis, and white fibrous rings were observed surrounding the migrated worms (Fig. 3C).

The ventral or visceral surface of the left lobe showed irregular patches of fibrosis and in the severely affected liver there was necrosis and abscess formation. Dilation of the bile ducts and congestion of the gallbladder can also be easily observed (Fig. 3D). In addition, both mature and immature *Fasciola* flukes can be seen in different regions of the liver. The bile ducts were dilated, and the portal lymph nodes were enlarged (Fig. 3E).

Cut sections, showed fibrotic thickening, calcification of bile ducts and massive hemorrhagic foci (Fig. 3F). In addition, bile ducts filled with debris and different stages of *Fasciola* were observed (Fig. 3G).

### Histopathological examination of non-infected sheep liver

**Figure 4:**
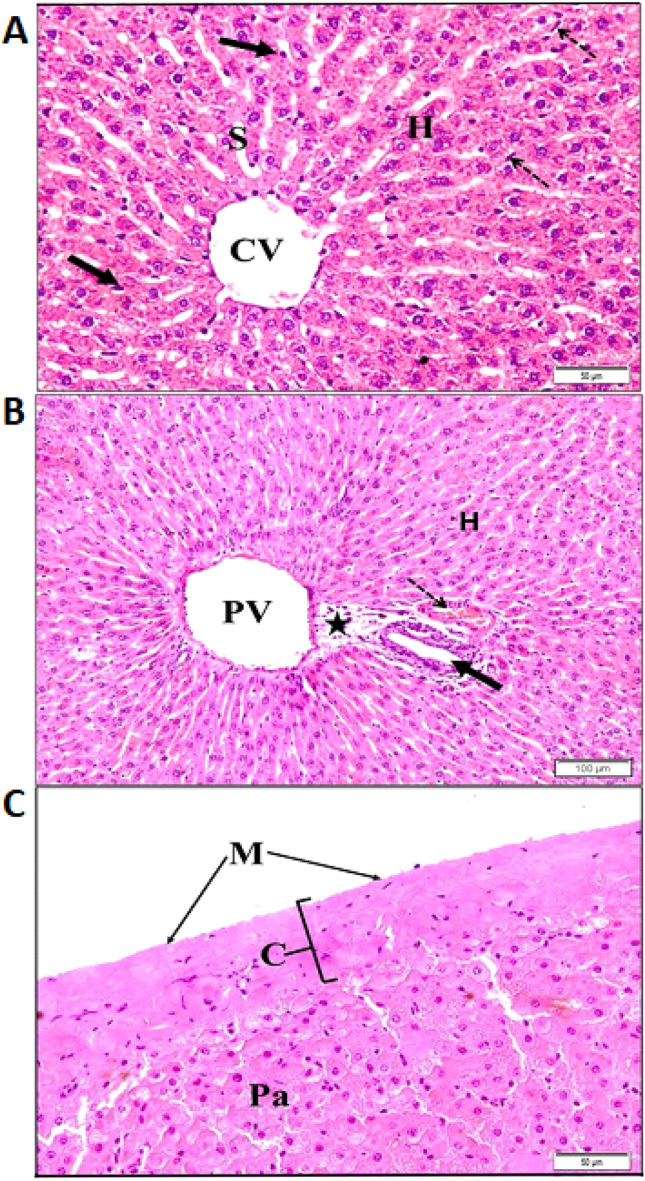
Light microscopic photographs of non-infected sheep liver stained with H&E. (**A**) Central vein (CV) surrounded by hepatocytes (H), separated by sinusoids (S), lined with endothelial (dotted arrows) and Kupffer cells (arrows); (**B**) portal region with portal vein (PV), bile duct (black arrow) and hepatic artery (dotted arrow) with scanty connective tissues (black star). Note the normal feature of hepatocytes (H); (**C**) intact normal parenchyma (Pa) and normal thin Glisson's fibrous capsule (C) covered by intact mesothelial cells (M).

The liver histology showed hepatocytes of normal size, rounded or polyhedral in shape. They have vesicular nuclei, and finely granular cytoplasm with intact outer lines. Hepatocytes appeared radiating outward from the thin-walled central vein (CV) receiving the blood from several sinusoids lined with endothelial and Kupffer cells (Fig. 4A). Portal regions showed normal portal vein (PV), and bile ducts lined by simple cuboidal to columnar epithelium. The connective tissue that surrounded the portal contents was scanty and showed few mononuclear inflammatory cells. Nearby hepatocytes and blood sinusoids showed normal histological features (Fig. 4B). In addition, the liver is surrounded by a thin fibroelastic capsule (Glisson’s capsule) showing collagenous tissue coated by a serous membrane of mesothelial cells (Fig. 4C).

### Histopathological examination of infected sheep liver

**Figure 5:**
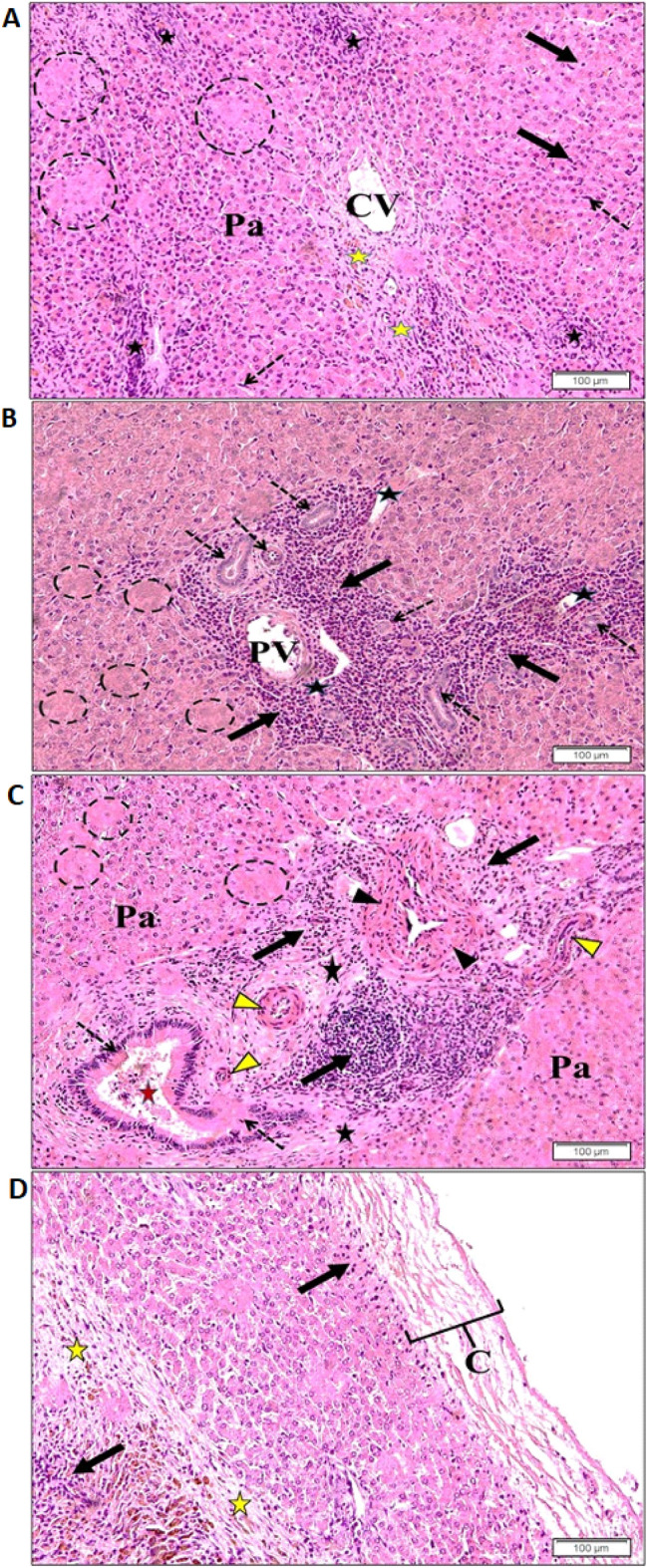
Light microscopic photographs of infected sheep liver stained with H&E. (**A**) Central vein (CV) region with disturbed parenchyma cells (Pa), focal lymphocytic infiltration (yellow stars), elongated endothelial cells (dotted arrows), enlarged Kupffer cells (arrow) and patches of lysed (necrotic) hepatocytes (dotted circle); (**B**) portal vein (PV) of the portal region with eosinophil and lymphocyte infiltration (arrows), the proliferation of bile ducts (dotted arrows) and lymphatic vessels (black stars). The adjacent liver parenchyma with patches of degenerating and lysed (necrotic) hepatocytes (dotted circles); (**C**) eosinophil infiltration, lymphocytes, and proliferating fibroblast (arrow), thickening of the hepatic artery (arrow heads) and arteriolar walls (yellow arrow heads), fibrosis (black stars), hyperplasia of bile ducts epithelium (dotted arrows) with damaged columnar epithelial and detached lining into the lumen (red star). The adjacent liver parenchyma (Pa) showed hepatocellular foci and lysis (dotted circles); (**D**) Glisson's thickened capsule (C), parenchyma fibrosis (yellow star) with sub capsular infiltrated inflammatory cells (arrows).

Several pathological features of *Fasciola* infection were observed in infected livers. In general, hepatocytes have a random distribution around the CV, sinusoids are compressed by swollen hepatocytes and appeared narrow, Kupffer cells are also large. The liver parenchyma in the central vein region showed necrotic perivascular hepatocytes, scattered patches of marked hepatocytes lysis, and aggregation of inflammatory cell infiltrate (Fig. 5A). Portal regions showed dilatation of lymphatic vessels and increased peri vascular connective tissue with fibrosis. Increased of bile duct proliferation and marked infiltration with eosinophils and lymphocytes were also observed (Fig. 5B). In addition, the portal region showed extensive fibrosis, marked hyperplasia of the bile ducts with damaged cellular endothelium, and thickening of the hepatic artery and arterioles walls (Fig. 5C).

**Figure 6:**
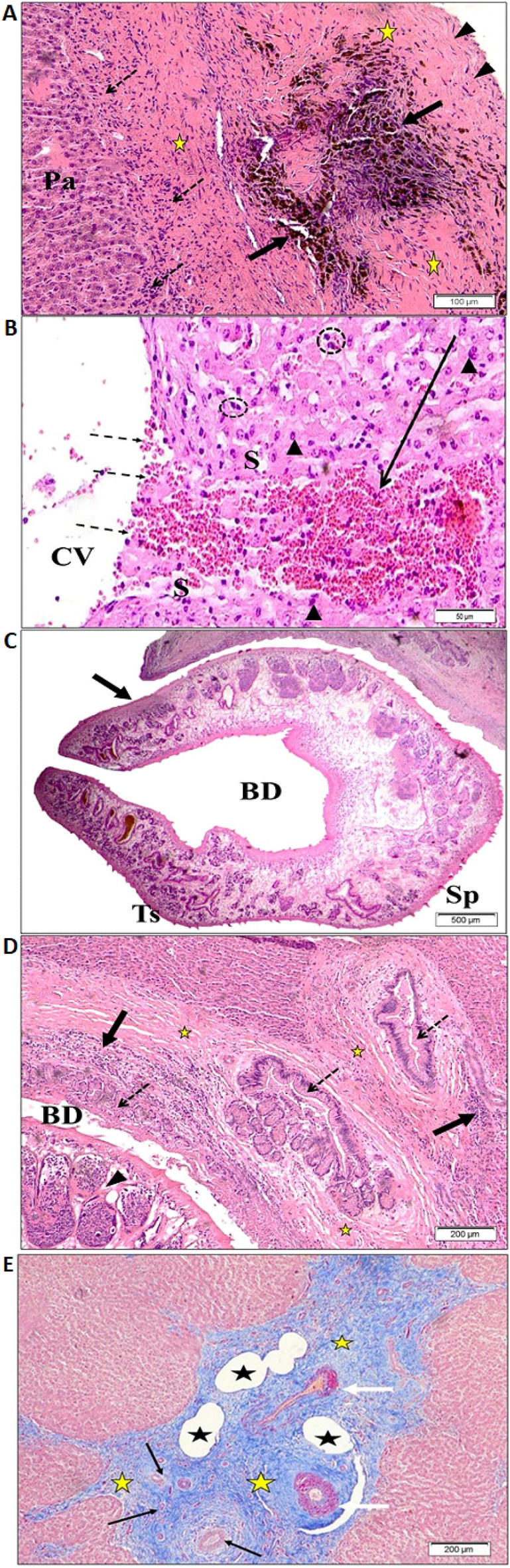
Light microscopic photographs of infected sheep liver stained with H&E. (**A**) thickened fibrous capsule (arrowhead) and capsule region of dark brownish hemosiderin pigment (arrows) with the marked proliferation of fibrous connective tissue (yellow stars). Inflammatory cells (dotted arrows) at the periphery of necrosed degenerated parenchymal hepatocytes (Pa); (**B**) part of dilated central vein (CV) with damage and loss of normal endothelial lining (dotted arrows). Note degenerated hepatocytes with dark stain nuclei (head arrows). Blood sinusoids showed enlarged Kupffer cells (dotted circle). Note massive hemorrhage at the site of necrotic hepatocytes (black arrow); (**C**) an adult fluke (arrow) in the lumen of the dilated bile duct (BD). Note the intact outer tegument (Ts) with embedded spines (Sp); (**D**) hyperplasia of the bile ducts (dotted arrows), extensive fibrosis of connective tissue (yellow stars) with heavy infiltration by various inflammatory cells (arrows). Note the presence of *Fasciola* worm (arrowhead) in the lumen of the dilated bile duct (BD). (**E**) Stained with Masson trichrome stain showing extensive fibrosis at portal region (yellow stars). Note the dilated lymphatic vessels (black stars), proliferated bile ducts (black arrows) and thickened walls of blood vessels (white arrows).

Furthermore, the Glisson’s capsule showed marked thickening with abnormal detachment of collagen fibers. Infiltration of inflammatory cells protruding from the capsule and throughout the parenchyma was observed with extensive fibrosis (Fig. 5C and Fig. 5D). Figure 6A showed marked dilatation of the central vein. The nearby parenchyma showed loss of normal hepatocyte shape and arrangement with dark stain nuclei, and massive hemorrhagic patches replacing necrotic hepatocytes could be observed. The hepatic sinusoids showed enlarged Van Kupffer cells (Fig. 6B).

### Histological features of adult *Fasciola* worm within portal area of infected sheep liver tissues

Mature adult flukes can be seen within the dilated bile ducts (Fig. 6C) that initiated various damaging effects including proliferation and hyperplasia of the bile ducts with infiltration of numerous inflammatory cells and increased fibrosis (Fig. 6D). The marked intensity of fibrous tissue in the liver sections was observed using Masson trichrome stain (Fig. 6E).

## Discussion

Due to the religious stance that Saudi Arabia enjoys, especially at the time of the Islamic pilgrimage (Hajj), the number of animals slaughtered increases dramatically. This increase in demand highly affects the prevalence of fascioliasis in the country and suggests much-needed control.

In 1987, Magzoub and Kasim^29^ were the first to report the prevalence of animal fascioliasis in Saudi Arabia. They found that the infection rates for sheep were 2.4%, 2.1% and 2.1% for the northern, eastern, and western regions, respectively. In our study, we observed that the rate of *Fasciola* infection was higher in imported slaughtered sheep compared to local breeding (2.12% and 0.67%, respectively).

A previous study evaluated the prevalence of fascioliasis among imported and local sheep in the Jeddah slaughterhouse. The infection within the imported sheep was 30.14%, while the local sheep were free of fascioliasis^30^. In Taif and Riyadh, comparative studies between imported and local slaughtered animals showed that the infection among imported sheep was higher than the local one^31,32^.

Recent prevalence rates of fasciolosis in sheep vary between countries around the world. Among the lowest rates were 2.37% in Sudan^33^, 4% in Switzerland^34^, 5.77% in Iraq^35^, 7.9% in Italy^34^, 13% in Iran^36^, 17.2% in Egypt^37^ and in Ethiopia^9^, 21.41%, in Pakistan^38^, and the highest rate (61.6%) was in Ireland^34^.

Effective fascioliasis control depends on good preventive measures to reduce definitive host infection by reducing intermediate hosts and controlling flocks and herd environments. To better apply such measures, information on seasonal variations and their relationship to animal infection must be obtained and evaluated. In our study, the highest infection among imported animals was in spring (2.95%) compared to summer, fall and, winter (2.67%, 2.25% and 1.26%, respectively), but without significant difference. This may be due to the uncontrolled contaminated feeding of animals in their original breeding grounds. The correlation between infection rate and seasons has been observed in several previously published studies conducted in different countries around the world^36,39–41^. The season with the highest infection rate varies from study to study, which can be attributed to the differences in climate and geographical topology.

In the present study, histopathological changes induced by *Fasciola* infection in sheep liver were examined using paraffin sections stained by H&E and Masson Trichrome for detection of fibrotic changes or collagen deposition. Histopathological examination in non-infected sheep liver showed hepatocytes of normal size, rounded or polyhedral shape. Hepatocytes and blood sinusoids also appear to have normal histological features. These features have been previously reported in the literature^42,43^.

Changes observed in the infected liver of sheep included hepatomegaly, change in the color of the liver, fibrosis, hemorrhage throughout the parenchyma, inflammatory changes, and abscess. These are known signs of fascioliasis that have been observed and reported previously^12,44,45^. The marked tissue damage was confined to the left lobe, located in the epigastric region of the abdominal cavity, and its lower surface protruding posteriorly and to the left of the gastric impression that forms on the surface of the stomach, and thus close to the migratory path of the fluke. In addition, the bile duct was specifically damaged in the infected liver, as it is the habitat for adult flukes. Signs such as hyperplasia, enlargement, calcification and thickening of the bile duct were also observed and confirmed by other studies^28,44^.

Glisson’s Liver capsule is composed of collagen, fibers with interspaced fibroblasts, and small blood vessels and coated by serous membrane^46^. In this study, Glisson’s capsule shows increased fibrous connective tissue proliferation leading to cirrhosis. Furthermore, hepatocytes showed a random distribution around the central vein, narrow sinusoids with elongation of their endothelial cells and enlarged Kupffer cells, evident fibrosis, in focal infiltrations with predominant lymphocytes and fibroblast, patches of cell-wall lysis where the edges of cells dissipate together, and the nuclei disappear. This indicates the acute phase or parenchymal phase of the infection with its subsequent pathological effects, which was fully consistent with previous studies^28,44^.

Our results are consistent with previous studies demonstrating that the migration of immature liver flukes through tissues irritates, and hemorrhage resulting in the aggregation of inflammatory cells^28,47^. It was also found that chronic fasciolosis leads to immunological reaction and infiltration of macrophages and lymphocytes that merge with fibrotic healing of the necrotic areas during the later phase of fasciolosis. These results were also reported by previous workers^48^.

Tissue atrophy and necrosis may be due to the digestion of host components by components by flukes through the release of proteases, facilitating their migration and feeding as well as immune evasion, which was consistent with MacGavin et al.^49^. In addition, eosinophilia has been recognized as a distinguishing feature of helminth infection in mammals and has been used as an important diagnostic measure for parasitic infection^50^.

In the present study, the bile ducts were visible, blocked by twisted flukes, and filled with hemosiderin due to an increased concentration of iron. This may be due to the presence of adult flukes within their lumen, which causes persistent irritation resulting in hyperplastic proliferations, enlargement, and extensive marred ductular fibrosis. The present finding is in line with the report of previous observations^17,28^. In addition, in agreement with previous studies, there were many inflammatory cells surrounding the fibrous connective tissue^17,44,45^.

Limitations of this study include the fact that we have not been able to study the risk factors related to the infection, as data were not made available to us. In addition, this study didn’t include molecular characterization of the isolated liver flukes due to lack of funding.

## Conclusions

The present study confirmed that the prevalence of fascioliasis is more common in imported sheep (2.12%) than in local breeds (0.67%). In addition, the current study demonstrated histopathological changes of the tegument of adult worms using H&E stain. Gross and histopathological hepatic changes induced resulting from *Fasciola* infection were also investigated to shed light on the detrimental effect of the infections on liver tissues and the defective nutritional value if consumed by humans, in addition to the fact that it may be a source of health risk.

This study raises attention to the importance of meat inspection records to monitor sources of disease and establish potential extended trends for such measures to reduce wastage and significant losses in the slaughter economy and to assist in the early identification of any parasitic diseases.

## Data Availability

All data generated or analyzed during this study are included in this published article.
